# Abdominal Wall Reconstruction in Abdominal Wall Endometriosis: A Case Report and Literature Review

**DOI:** 10.1055/a-2336-0073

**Published:** 2025-03-11

**Authors:** Otis C. van Varsseveld, Gustavo G. Koeijers, Juan M. Rodriguez Vitoria, Igor Gomes Bravio

**Affiliations:** 1 Department of Surgery, University Medical Center Groningen, University of Groningen, Groningen, The Netherlands; 2 Department of Plastic, Reconstructive and Hand Surgery, Curaçao Medical Center, Willemstad, Curaçao; 3 Department of Surgery, Curaçao Medical Center, Willemstad, Curaçao; 4 Department of Obstetrics & Gynecology, Curaçao Medical Center, Willemstad, Curaçao

**Keywords:** surgery, plastic, abdominoplasty, endometriosis, surgical mesh, abdominal wall

## Abstract

Abdominal wall endometriosis (AWE) is a rare condition representing 1% of patients operated for endometriosis. We describe a case of a 26-year-old woman, with a history of cesarean delivery, who presented with cyclical pain and a subcutaneous mass in the lower abdomen. Where most AWE lesions may be surgically managed by a single surgeon, imaging revealed an unusually large lesion (13 × 4 × 10 cm) involving the rectus abdominis muscle. Plastic, gynecologic, and general surgeons combined their expertise to conduct AWE excision combined with miniabdominoplasty in a single procedure. After resection, a retrorectus mesh (Rives–Stoppa technique) reinforced the primarily closed posterior rectus sheath and an inlay mesh bridged the defect left in the anterior rectus sheath. The patient was discharged 3 days postoperatively, had minimal pain complaints, and was satisfied with cosmetic results at 1-month and later follow ups. One year postoperatively, she gave uncomplicated vaginal birth. We conclude that, in select cases, management of a large, symptomatic AWE may benefit from a multidisciplinary approach, where symptom relief and an aesthetically pleasing result for the patient can be achieved in a single procedure. We distinctively describe double mesh repair as a viable consideration for reconstruction in AWE and review current considerations in mesh repair of the abdominal wall. Further studies into this topic are warranted.

## Introduction

Abdominal wall endometriosis (AWE) is a rare condition in which glands and stroma of endometrium are encountered within abdominal wall layers. The prevalence of AWE is reported at 1.3% in patients operated for endometriosis and represents just 4.8% of extrapelvic endometrioses. 1 2 AWE is associated with a cesarean scar, occurring in 0.03 to 1% of women who have undergone cesarean delivery. 3 4 Patients mostly present with an abdominal wall mass (96%) and pain (87%), which may be concurrent with the menstrual cycle (57%). 3

Surgical excision is considered the standard of care for AWE, with a recommended excision margin of 1 cm. 5 Conservative hormonal treatment is found to give only temporary relief from symptoms. 5 Considering an average greatest dimension of 2.7 cm, most AWE lesions may be managed by excision and primary fascial closure by a gynecologic or general surgeon. 3 5 Yet, lesions over 3 cm or involving the rectus muscle may require consultation of multiple specialists. 4 In this case report, we present a combined effort between plastic, general, and gynecologic surgeons for the management of an unusually large AWE. Written informed consent was obtained from the patient prior to conception of the manuscript.

## Case

**Fig. 1:**
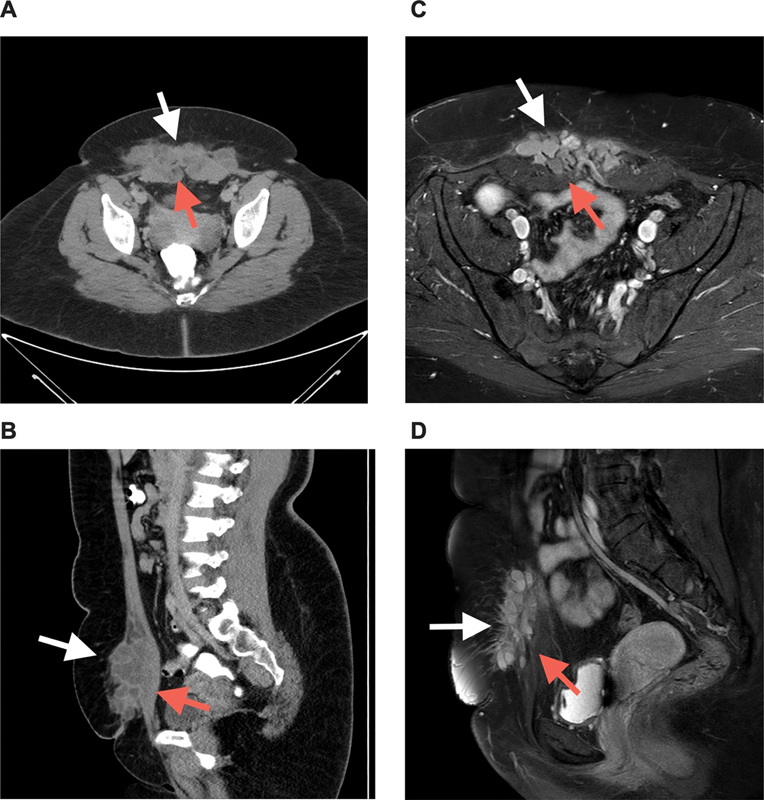
( **A, B** ) Axial and sagittal abdominal CT with contrast 2 weeks after initial presentation at the Gynecology department, showing a mass in the subcutis (white arrows) and inferior part of the rectus muscles (red arrows). ( **C, D** ) Axial and sagittal abdominal magnetic resonance imaging at 7 months after neoadjuvant treatment with monthly leuprorelin injections, showing a mass unchanged in size (white arrows) with involvement of the medial part of the rectus sheath and muscles (red arrows).

A 26-year-old woman with a history of cesarean delivery and a body mass index (BMI) of 32.4 kg/m ^2^ , was referred to the Department of Gynecology with a suspicion of endometriosis. A subcutaneous mass at the level of her nearly 3-year-old Pfannenstiel scar had been present for 2 years with pain subsiding and worsening along with her menstrual cycle. Upon physical examination, there was no superficially visible mass around the Pfannenstiel scar, but palpation revealed painful subcutaneous lumps. Computed tomography with contrast of the abdomen showed an anterior abdominal wall lesion congruent with endometriosis measuring 13 × 4 × 10 cm ( Fig. 1A, B ). Ultrasonography-guided needle aspiration confirmed the presence of endometrial micropolyps in the lesion. Monthly leuprorelin injection was opted as neoadjuvant therapy to reduce the lesion size preoperatively but provided only mild pain relief and no reduction in endometriosis mass at 7 months ( Fig. 1C, D ). Magnetic resonance imaging showed an unchanged lesion with involvement of the rectus abdominis muscle and fascia. A multidisciplinary surgical approach was planned with a plastic and reconstructive surgeon, general surgeon, and gynecologist to ensure safety, efficacy, and cosmesis.

An ellipse-shaped miniabdominoplasty marking was made between approximately 5 cm below the umbilicus and the level of the Pfannenstiel scar. Incision started cranially, resecting till the rectus sheath, and was then continued on the caudal side of the ellipse extending the Pfannenstiel scar along the bikini line. Abdominal wall tissue was mobilized from the rectus fascia laterally to medially until the endometrial tissue was encountered. A single block of skin, subcutaneous tissue, and endometriosis was resected, exposing the endometrial tissue affecting the central part of the rectus abdominis sheath and muscles.

The rectus sheath was incised near the midline close to the medial junction of the anterior and posterior rectus sheath to preserve the posterior sheath area. The posterior rectus sheath was separated from the muscle by retrorectus dissection up to the lateral junction with the anterior sheath, preserving neurovascular bundles not involved in the lesion. Retrorectus dissection was continued caudally along the extent of the endometriosis, bilaterally, reaching past the arcuate line and exposing the retropubic (Retzius') space. After resection of endometrial tissue, a defect with a variable width of 4 to 6 cm was left in the posterior rectus sheath, whereas a larger defect of approximately 14 × 11 cm was left in the anterior rectus sheath. The depth of the defect focally extended into the parietal peritoneum.

**Fig. 2:**
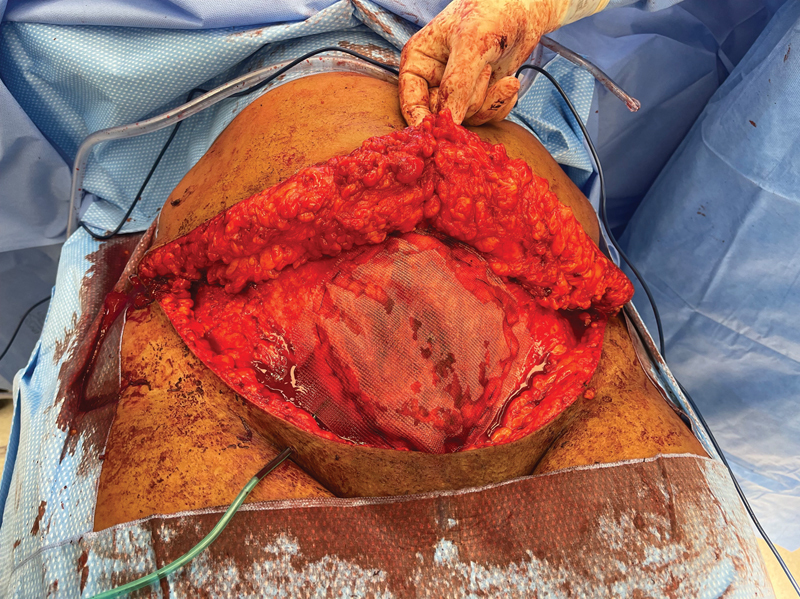
Intraoperative picture displaying the inlay polypropylene mesh bridging the anterior rectus sheath defect caused by excision of the abdominal wall endometriosis, and the mobilized and partially resected abdominal wall skin and subcutaneous tissue for miniabdominoplasty.

The posterior rectus sheath was primarily closed, together with the focal peritoneal defect, with delayed absorbable sutures. Subsequently, retrorectus (sublay) placement (Rives–Stoppa technique) of a nonabsorbable, macroporous mesh was performed, which was fixated onto the pectineal (Cooper's) ligaments and the posterior sheath. Upon closure of the anterior rectus sheath, the defect had to be bridged by a polypropylene inlay mesh ( Fig. 2 ). Drains were placed and the abdominal wall was further closed in layers, subcutaneously and cutaneously, with absorbable sutures. Postoperatively, cefazoline antibiotic prophylaxis was continued for 72 hours and drains were removed upon producing less than 50 mL in 24 hours—on the third postoperative day. An abdominal binder was worn by the patient for 2 weeks.

**Fig. 3:**
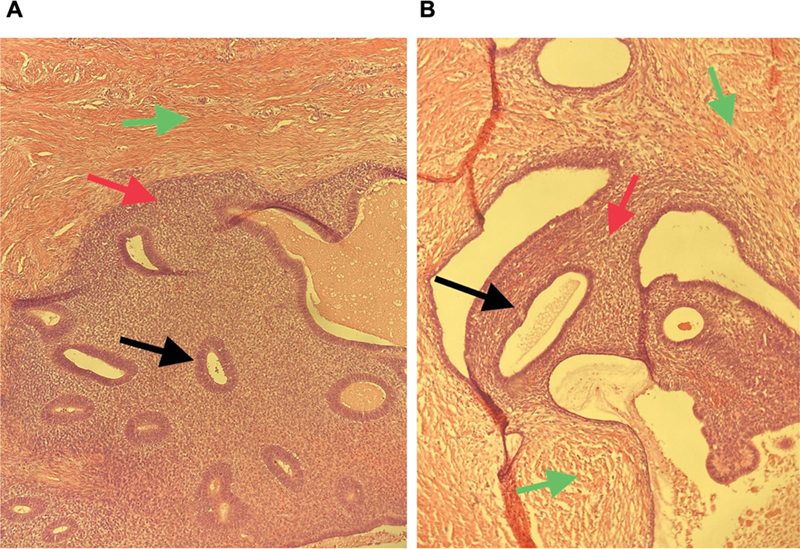
Pathology slides showing the fibroadipose tissue (green arrows) of the abdominal wall with an overgrowth of endometrial glands formed by cylindrical cells (black arrows). Endometrial glands are surrounded by endometrial stroma (red arrows).

**Fig. 4:**
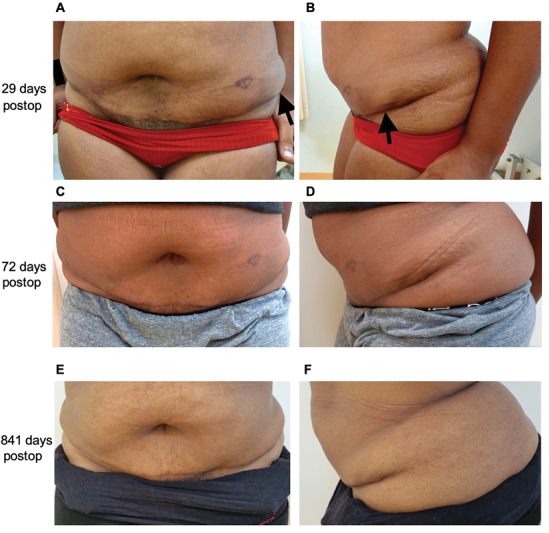
Postoperative results. ( **A, B** ) display the miniabdominoplasty wound at 29 days postsurgery where a seroma can be seen on the left side of the scar. By 72 days postsurgery ( **C, D** ), the seroma was reabsorbed spontaneously. ( **E, F** ) show the scar at 2.5 years follow-up.

Pathology of the excised tissue confirmed extensive endometriosis with no signs of malignancy ( Fig. 3 ). The postoperative course was uncomplicated with drain removal and discharge 3 days after surgery. One month postoperatively, the patient had minimal pain complaints and was satisfied with the cosmetic results of the surgery. A self-limiting seroma occurred at the left end of the miniabdominoplasty scar that was reabsorbed by the 2-month follow-up ( Fig. 4 ). At 6 months, 1-, and 2.5-year follow-up, the patient remained free from pain complaints and the AWE did not recur. One year and 3 months after the surgical procedure, she gave uncomplicated vaginal birth to a healthy child.

## Discussion

In this case report, we described a challenging presentation of the rare condition AWE. By an integrated, multidisciplinary approach between a plastic and reconstructive surgeon, a general surgeon, and a gynecologist, the lesion was safely removed with an adequate abdominal wall reconstruction. This was effectively combined with miniabdominoplasty in a single procedure to yield an additional cosmetic result.

**Fig. 5.**
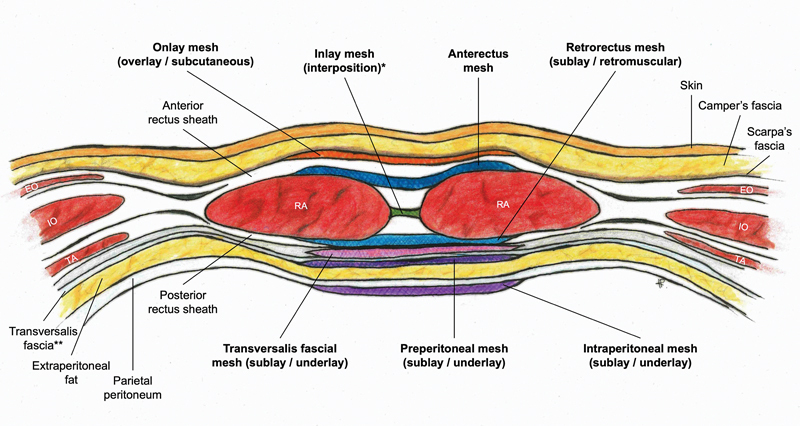
Illustrated axial cross-section of the anterior abdominal wall above the arcuate line displaying different planes of surgical mesh insertion. Plane names are in accordance with international consensus-based definitions of Parker et al (2019). 10 Colloquial, interchangeably used names are shown between parentheses. *By definition, any bridging mesh, regardless of anatomical plane, is called an inlay or interposition mesh. In our case report, the retrorectus (sublay) mesh reinforced the primarily closed posterior rectus sheath, and the second inlay (interposition) mesh was positioned as a bridge between the two sides of the large anterior rectus sheath defect. **In this illustration, transversalis fascia was depicted as separated from the posterior rectus sheath, demonstrating the potential space for the transversalis fascial mesh plane. EO, external oblique muscle; IO, internal oblique muscle; RA, rectus abdominis muscle; TA, transversus abdominis muscle. Illustration by Ilse I. Posthumus.

With a reported incidence of up to 20%, incisional hernias after laparotomy are a major consideration for choosing mesh placement in abdominal wall reconstruction. 6 7 Even though herniorrhaphy with mesh placement is a common and well-studied procedure, the nomenclature of the different abdominal wall planes for mesh insertion remains ambiguous. 8 Most commonly, the terms onlay, inlay, and sublay or underlay are used, whereas their specific definition may still differ. 8 9 10 11 In 2019, Parker et al 10 published an international consensus-based classification for abdominal wall planes to improve communication and comparison among surgeons and research studies. The anatomical planes for mesh insertion are schematically displayed in Fig. 5 .

The ideal plane for mesh insertion has been debated widely in the past and practice still varies. However, two comprehensive systematic reviews, together including over 12,000 patients, have shown that recurrence rates are lower in retrorectus and “underlay” (including preperitoneal and intraperitoneal) mesh placement compared to onlay and inlay mesh placement for abdominal wall reconstruction. 6 11 It was also proven that in abdominal wall reconstruction, primary fascial closure combined with preperitoneal (underlay) mesh reinforcement should be preferred over fascial bridging with mesh to reduce hernia recurrence. 12 In the reported case, we opted for retrorectus (sublay) mesh reinforcement ( Fig. 5 ), which was shown to have the lowest recurrence and infection rates compared to other mesh placements and good long-term outcomes in abdominal wall repair. 6 11 13

We were forced to place a second mesh for bridging—called “inlay” mesh by definition as it bridges a defect, regardless of plane 9 10 —because the residual anterior rectus sheath defect was too large to close primarily ( Fig. 5 ). Double mesh abdominal wall repair is less extensively studied, as the technique is mostly reserved for large, complex incisional hernias. 14 Reoccurrence rates seem to be more favorable compared to single mesh repair but wound complications seem to be more frequent. 14 15 Yet, the quality of the evidence is still low and insertion plane and mesh type vary widely. 14 Moreover, these studies are not directly applicable to our case as this concerns a primary reconstruction. Fortunately, our patient remained infection- and recurrence-free at 1-year follow up. She did suffer from a self-limiting seroma, but this is not uncommon considering an incidence of 11% after abdominal wall reconstruction with mesh. 11

Another variable that should be considered in abdominal wall reconstruction is mesh type. Different materials may be used, including synthetic permanent mesh, synthetic absorbable mesh (short-term, and long-term “biosynthetic mesh”), synthetic composite mesh (both permanent and absorbable components), and biological mesh (acellular collagen matrices). 16 The choice for a specific mesh remains surgeon- and case-dependent. Earlier systematic reviews and a recent meta-analysis have shown that reconstruction with biological mesh gives higher hernia recurrence rates and surgical site infection rates compared to synthetic mesh. 6 11 16 17 Still, it must also be noted that traditionally biological mesh is opted in larger and/or contaminated abdominal wall repairs, which likely influenced its complication rate in nonrandomized studies. 9 11 17 In our described case, the synthetic permanent mesh was used as no alternatives were at hand nor indicated in accordance with our assessment.

Finally, the extent the defect that has to be reconstructed should be an important consideration in abdominal wall reconstruction. The extent of abdominal wall involvement can be divided into different abdominal wall regions and tissue depths, as was done in the M.D. Anderson Oncological Abdominal Wall Reconstruction Classification. 18 M.D. Anderson grade V involves two or more of the classified abdominal wall regions—in our case region I and IV, above and below the arcuate line, respectively—and often does not allow primary fascial reapproximation. 18 Concordantly, in our patient a synthetic mesh had to bridge the anterior rectus sheath defect.

Combining AWE resection with a plastic surgery technique such as (mini-) abdominoplasty may be aesthetically valuable to the patient. In this case, where AWE involved both subcutaneous and musculofascial tissue, it also allowed the required wide excision margin for the lesion without major issues of subcutaneous and cutaneous approximation. 5 Despite the “double” surgery, our patient benefitted from a good recovery time with a length of stay of 4 days compared to conventional mesh repair (10–14 d) or conventional abdominoplasty (4.5–6.7 d). 7 19 Hence, this combined approach allowed us to tackle pain symptoms and cosmetic wishes in one intervention for this specific patient. Although this combined surgical approach with double mesh repair for the rare condition AWE has not been studied to our knowledge, a small case series with miniabdominoplasty and single mesh repair corroborates the safety of this procedure. 20 Nevertheless, further studies are warranted.

### Conclusion

Management of large, symptomatic AWE lesions involving the subcutaneous tissue and rectus muscle should be conducted by a multidisciplinary team of specialists familiar with endometriosis and abdominal wall reconstruction. When an aesthetic result has priority for a patient, a combination of AWE resection and abdominal wall reconstruction with (mini-) abdominoplasty is a viable option. Considerations in the abdominal wall reconstruction should include (double) mesh placement, mesh type, and the extent of the lesion.
